# Understanding Foot Loading and Balance Behavior of Children with Motor Sensory Processing Disorder

**DOI:** 10.3390/children9030379

**Published:** 2022-03-09

**Authors:** Lin Yu, Peimin Yu, Wei Liu, Zixiang Gao, Dong Sun, Qichang Mei, Justin Fernandez, Yaodong Gu

**Affiliations:** 1 Faculty of Sports Science, Ningbo University, Ningbo 315211, China; yulin713@hotmail.com (L.Y.); pyu926@aucklanduni.ac.nz (P.Y.); liuwei2@nbu.edu.cn (W.L.); gaozixiang0111@outlook.com (Z.G.); j.fernandez@auckland.ac.nz (J.F.); 2 Faculty of Sports Sciences and Coaching, Sultan Idris Education University, Tanjong Malim 35910, Malaysia; 3 Research Academy of Grand Health, Ningbo University, Ningbo 315211, China; 4 Auckland Bioengineering Institute, University of Auckland, Auckland 1010, New Zealand; 5 Faculty of Engineering, University of Pannonia, 8200 Veszprém, Hungary; 6 Department of Public Service and Management, Ningbo College of Health Sciences, Ningbo 315199, China

**Keywords:** children gait, sensory processing disorder, plantar loading, center of pressure, foot balance, SPM1D

## Abstract

Sensory processing disorder (SPD) could influence the neuromuscular response and adjustment to external sensory discrimination and lead to disruptions in daily locomotion. The objective of the current study was to compare plantar loadings and foot balance during walking, running and turning activities in SPD children in order to reveal the behavioral strategy of movement and balance control. Six SPD children and six age-match healthy controls participated in the test using a FootScan plantar pressure plate. The time-varying parameters of forces, center of pressure and foot balance index were analyzed using an open-source one-dimensional Statistical Parametric Mapping (SPM1d) package. No difference was found in foot balance and plantar loadings during walking, while limited supination–pronation motion was observed in the SPD children during running and turning. The plantar forces were mainly located in the midfoot region while less toe activity was found as well. Findings should be noted that SPD children had limited supination–pronation movement for shock attenuation in the foot complex and reduced ankle pronation to assist push-off and toe gripping movements. Understanding the behavior of plantar loading strategy and balance control during walking, running and turning activities may provide clinical implications for the rehabilitation and training of daily tasks.

## 1. Introduction

Sensory processing disorder (SPD) is affecting more and more school children, as documented by a ratio of around 8.3% in a recent study [1,2]. SPD often accompanies other symptoms, such as autism and attention deficit hyperactivity disorder (ADHD). SPD may result from preborn or postborn factors [3,4]. This disorder is reported as an neurological impairment that limits the brain’s ability to receive, process and respond to sensory stimuli [3,5,6]. The prevalence of SPD has increased in school-aged children and children that are firstly diagnosed [7,8]. Children with SPD may have problems responding to sensory discrimination, thus resulting in disruptions such as high risks of falling during dynamic activities in daily life.

A recent study classified the SPD into three main categories, including sensory modulation disorder, sensory discrimination disorder and sensory-based motor disorder [9]. The sensory modulation disorder could be further categorized into three subtypes includes over-responsivity, under-responsivity and seeking/graving [9]. Sensory discrimination disorder, as second category of SPD, typically refers to the difficulty in interpreting the specific characteristics of sensory stimuli occurring in auditory, visual, gustatory, vestibular, olfactory, tactile and proprioceptive systems [10]. As for sensory-based motor disorders, two subcategories (postural disorder and dyspraxia) are proposed. Postural disorder might have difficulties in balance control and core stability, and dyspraxia is related to motor planning as well as sequencing movements [9,11].

Daily locomotion often involves the activities of walking, running and turning, which are demanding automatic motor skills. The gait pattern is based on the combination of multiple factors including mechanical, neurological, cognitive and perceptual factors [11]. Compared to the walking gait, running and turning tasks are also employed apart from during daily activities and many sports. The more dynamic running and turning locomotion are, the more challenging the integrative aspects of mechanics, cognition and physiology [12,13]. The walking gait is the pendulum mechanism, while running is a spring-loaded mechanism [14]. The turning (or cutting maneuver) consists of a quick deceleration of the body and acceleration in the new movement direction [15]. Adjustments of the body position and acceleration are required when changing from the initial direction, accompanying alterations in several parameters, such as ground reaction forces and center of pressure (COP) [16,17].

The plantar pressure measurement has been widely employed to analyze functions in the foot and ankle complex during functional and daily life activities, and it is considered as an important clinical indicator while evaluating patients with musculoskeletal, integumentary and neurological disorders [18,19,20,21]. A comprehensive understanding of the plantar pressure distribution is vital to detect anatomical foot deformities, thus further providing prevention strategies and personalized exercise treatment programs [22,23,24]. Plantar pressure and gait balance are also used in detecting the postural stability in the population with sensory processing problems [6,25]. Kim et al. employed COP and plantar pressure to detect the balance deficit in children with attention-deficit/hyperactivity disorder (ADHD) [25]. In a recent study about the children with autism, the altered plantar pressure variables (time to peak pressure and distribution of the integrated pressure) indicated that these children showed abnormal gait patterns compared to healthy counterparts [26]. Plantar pressure has been proved as a clinical useful outcome measurement to detect and quantify the gait characteristics of children with cerebral palsy [27]. Plantar pressure and COP trajectory are also used to detect the influence of external strap orthosis on children with mild cerebral palsy [28].

Recent studies have investigated the plantar pressure (loading) distribution of the population with developmental disorders such as autism, ADHD and cerebral palsy. However, there is a lack of literature on the foot balance and force distribution in children with SPD, especially during basic daily locomotion (walking, running and turning activities) compared to the typically developing healthy cohort. Understanding the plantar loading characteristics and foot balance of the children with SPD may assist understanding the strategy of movement and balance control, thus providing clinical implications for the rehabilitation and training of daily tasks. Accordingly, the aim of this study is to compare the differences in the plantar loadings, foot balance and COP trajectory of children with SPD when executing walking, running and turning tasks in both dominant and non-dominant feet. It is hypothesized that children with SPD would alter plantar loading in foot regions and present reduced foot balance during high dynamic running and turning activities.

## 2. Materials and Methods

### 2.1. Participants

A total of twelve male children participated in this study, including six children with SPD (age: 5.83 ± 1.17 years, height: 112.33 ± 7.76 cm, weight: 19.5 ± 4.37 kg) and six healthy controls (age: 7.1 ± 1.2 years, height: 134.2 ± 5.7 cm, weight: 24.1 ± 3.1 kg). The six SPD children have postborn acquired symptoms and were diagnosed into the category of the sensory-based motor disorder from the clinical physical therapist [9], following a previously established Children’s Sensory Integration Development Rating Scale [29,30,31]. All participants were free of lower limb injuries or surgeries within half a year prior to the test. The dominant limb of each participant is the right leg, defined as the preferred foot for ball kicking [32]. Prior to data collection, all participants were informed of the procedures about the experimental protocol and signed consent forms. This study was approved by the Human Ethics Committee in the Research Institute of Ningbo University (RAGH20201216).

### 2.2. Test Protocol

**Figure 1:**
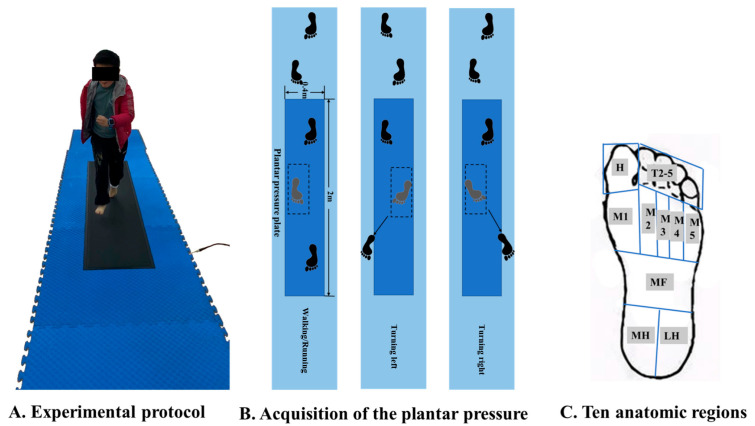
Illustration of the test protocol of straight running (**A**) and left and right turning (**B**) and division of anatomic regions (**C**).

Foot pressures during walking, running and turning activities were measured via a FootScan plantar pressure plate (RsScan International, Olen, Belgium) with dimensions of 2 m × 0.4 m × 0.02 m with 16,384 sensors at the sample frequency set as 480 Hz, which is embedded in the 20 m runway. Before formal experimentation, the weight of each participant was measured to calibrate the pressure plate, following a protocol employed in our previous studies [19,33]. Furthermore, all participants were given five minutes to warm up and familiarize with the test environment and requirements. The walking, running and turning right (with left foot) and turning left (with right foot) tasks were included in this study (Figure 1A,B), and all tasks were conducted under the barefoot condition.

To record normal and actual locomotion performance, all participants were required to walk, run and turn at a self-selected speed. When turning right, the stance leg of left foot was analyzed. Accordingly, when turning left, the right foot was measured. Trials of incomplete step on the pressure plate would be excluded. Three successful trials of both limb from each participant were recorded during walking, running and turning tasks, respectively. Foot pressure on ten anatomic regions was further processed and analyzed, which included Toe 1 (H), Other Toes (T2-5), Metatarsals I-V (M1–M5), Midfoot (MF), Medial heel (MH) and Lateral heel (LH) (Figure 1C). The foot pressure parameters were processed by one experienced assessor in the RsScan software system (RsScan International, Olen, Belgium).

### 2.3. Data Processing

**Figure 2:**
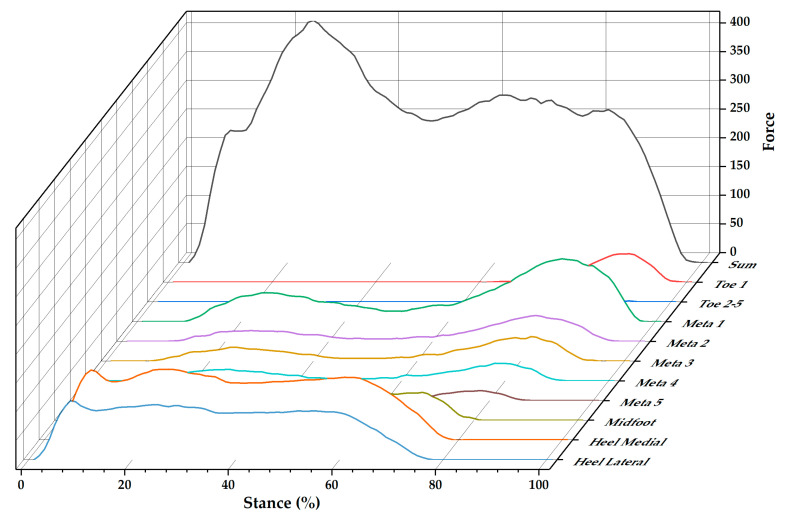
Example of regional and sum ground reaction forces during walking.

The time varying features of sum and regional ground reaction forces during stance are shown in Figure 2. The regional forces are selected with force values with an exclusion of zero. Then, the time series of force data from stance foot during walking, running and turning tasks were processed and interpolated to a length of 51 data points via a cubic spline [34].

The calculation of foot balance index (***FBI***) is based on the mean value of peak pressure in metatarsal joints and the heel regions, which is presented in Equation (1). ***FBI*** presents total foot stability with the positive value indicating foot pronation and the negative value indicating foot supination [28,33]:

(1) $$FBI=\frac{\left(M1+M2+MH\right)-\left(M3+M4+M5+LH\right)}{F_{avg}}*100\%$$

where ***F_avg_*** represents the average value of the entire plantar force.

The COP trajectory is one of the key parameters to reveal gait characteristics [20,21,35,36]. In this study, the COP and ***FBI*** trajectory over the stance of walking, running and turning activities were also analyzed and interpolated to a uniform length of 51 points via a cubic spline for statistical analysis.

### 2.4. Statistical Analysis

Three trials of walking, running and turning each were firstly averaged as one to represent this participant, thus avoiding intertrial variations. As per the previously reported protocol [19,37,38], the time series of force is normalized by ***Z_avg_***, which is calculated by using the total force divided by the sum of original data frames. For the one-dimensional time-varying parameters (time-series forces, COP and FBI), the normal distribution is firstly checked and statistical analysis is then evaluated using the independent sample t-test package in statistical parameter mapping 1d (SPM1d) based on the random vector filed theory [34,39]. The time series data were assessed in MATLAB R2018a (The MathWorks, MA, USA). The variables are presented by mean and standard deviation (SD). A two-tailed confidence interval with a *p* value of less than 0.05 was applied to define statistically significant results.

## 3. Results

This study analyzes the center of pressure trajectory, foot balance index, ground reaction force and regional plantar forces in the left and right foot during walking, running and turning activities. In this section, the main results with significant difference are presented, while other information is included in Supplementary Materials for reference.

### 3.1. Center of Pressure Trajectory

The COP trajectory shows no significance between SPD children and healthy controls, apart from the push-off phase around 92–95% of stance in the right foot. Full information is illustrated in the Figure S1. It also could be noted that the left foot is in a supination position, while the right foot lands in a neutral foot position until push-off.

**Figure 3:**
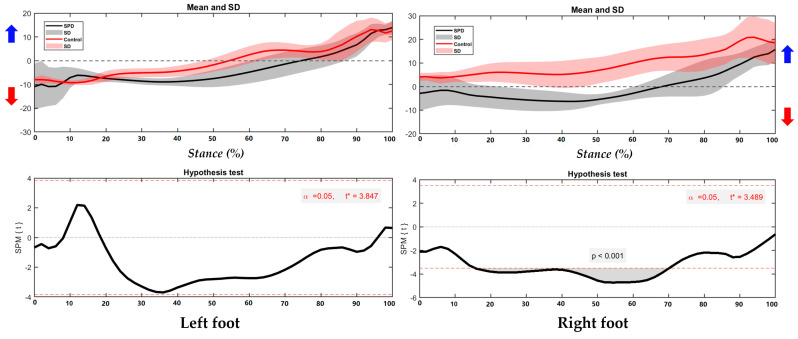
The COP of trajectory in the left foot and right foot during running with highlighted direction of pronation (Blue arrow) and supination (Red arrow).

As shown in the Figure 3, the right foot shows significant different foot position in the COP trajectory, especially during 16–69% of stance (*p* < 0.001).

**Figure 4:**
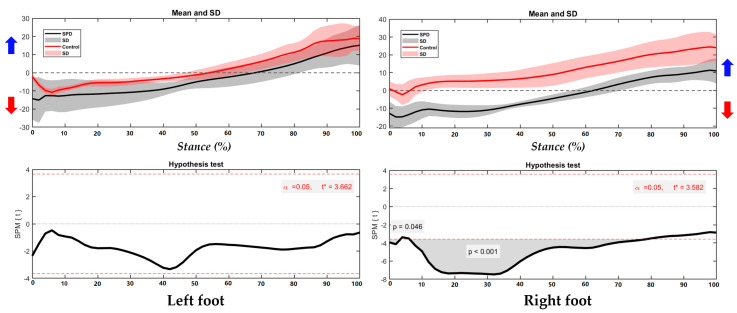
The COP of trajectory in the left foot and right foot during right/left turning with highlighted direction of pronation (blue arrow) and supination (red arrow).

As shown in the Figure 4, the right foot presents significantly different foot position in the COP trajectory, particularly during 0–4% and 7–74% of stance. The left is similar with running without significance between SPD children and healthy controls.

### 3.2. Foot Balance Index

**Figure 5:**
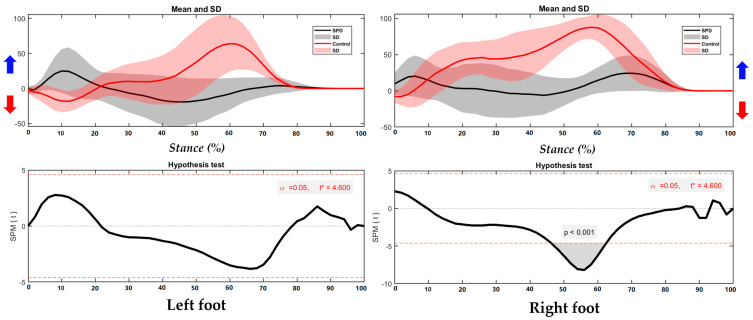
FBI in the left foot and right foot during running with highlighted directions of pronation (blue arrow) and supination (red arrow).

The foot balance index (FBI) of the left and right foot shows no significance between the SPD children and healthy controls (refer to the Figure S2 for full information). While the left and right foot had similar trends of foot balance index during running in SPD children with limited supination–pronation range, while during push-off, the healthy children show an obvious pronation trend in the FBI (Figure 5), particularly in the right foot where statistical significance is found during 47–63% of stance (*p* < 0.001).

**Figure 6:**
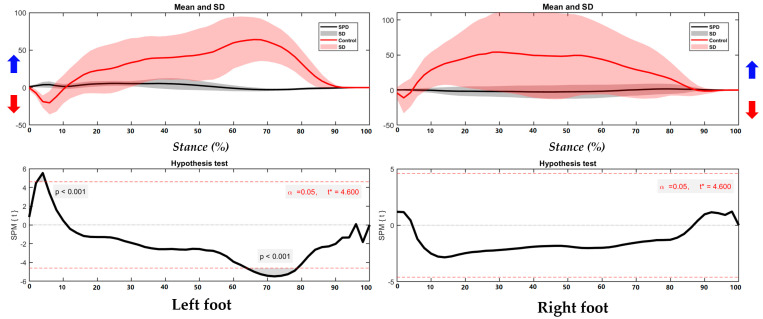
FBI in the left foot and right foot during turning with highlighted direction of pronation (blue arrow) and supination (red arrow).

Due to the high dynamic nature of turning motion, healthy children showed supination–pronation shifts in both feet, whereas SPD children presented restricted supination–pronation during both left turning with right foot and right turning with left foot (Figure 6). Significance is observed in the left foot during initial landing and the push-off phase.

### 3.3. Ground Reaction Force and Regional Plantar Forces

The ground reaction force in the left and right foot of SPD children and healthy controls showed no significance during walking (refer to the Figure S3 for full information), and regional plantar force presented no significance during walking (refer to the Figure S4 for information).

**Figure 7:**
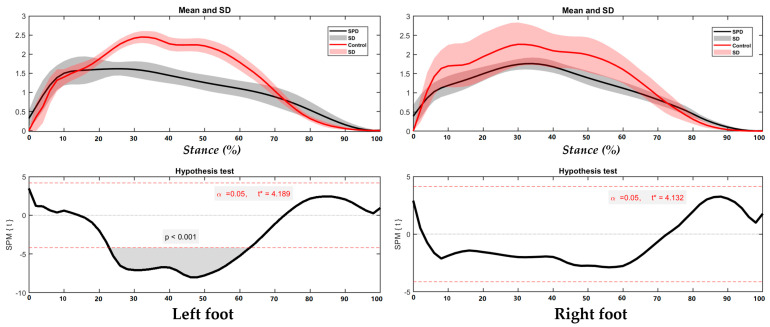
The vertical ground reaction force (GRF) in the left foot and right foot during running in SPD children and healthy controls (unit: ***Z_avg_***).

The magnitudes of running GRF are higher in healthy controls than SPD children, especially in the left foot where healthy controls are significantly larger than SPD children from 22–63% during stance. Similar magnitudes were observed in the right foot, although no significance is found (Figure 7).

**Figure 8:**
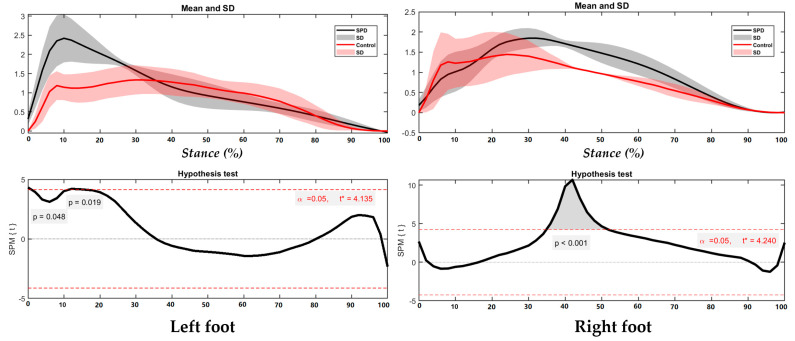
The vertical ground reaction force (GRF) in the left foot and right foot during turning in the SPD children and healthy controls (unit: ***Z_avg_***).

Contrary results are observed during turning (or cutting) motions with the left and right foot. The left foot of SPD children shows greater GRF than the healthy control during initial landing (1–2% and 11–15%) phase while turning right. The right foot of SPD children shows larger GRF around the mid stance (35–52%) and afterwards, although without significance (Figure 8).

**Figure 9:**
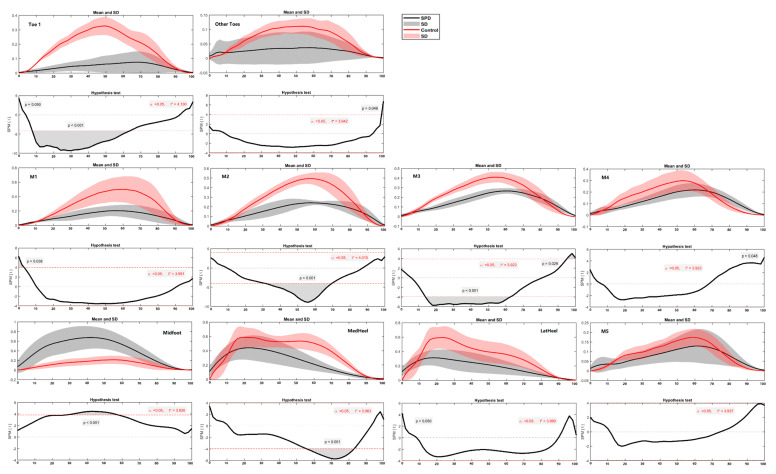
The regional plantar forces in the left foot during running in the SPD children and healthy controls with highlighted statistics.

**Figure 10:**
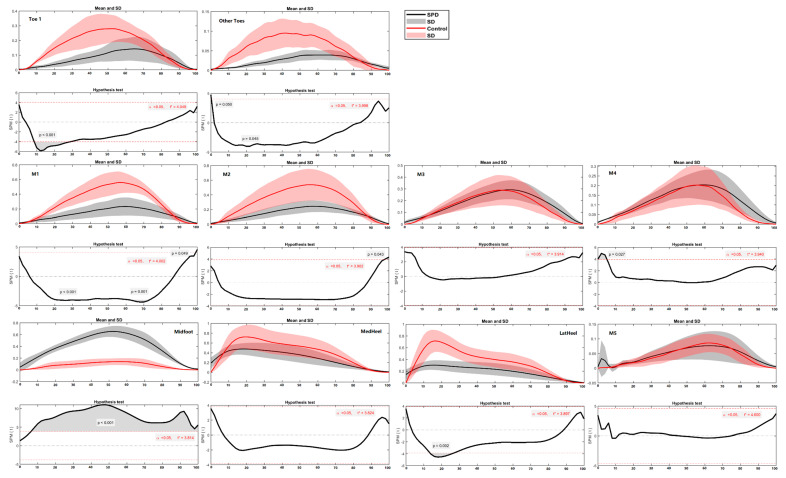
The regional plantar forces in the right foot during running in the SPD children and healthy controls with highlighted statistics.

In terms of the regional plantar force during running, the differences between SPD children and healthy controls were mainly found in the toe (Toe 1 and Other Toes), medial forefoot (M1, M2 and M3) and midfoot regions (Figure 9 and Figure 10) during certain phases (in percentage) of the stance. Specifically, healthy controls had greater force in Toe 1 (during 7–62%, *p* < 0.001), Other Toes (during 98–100%, *p* = 0.049), M1 (during 1–4%, *p* = 0.038), M2 (during 33–69%, *p* < 0.001), M3 (during 14–62%, *p* < 0.001) and MedHeel (during 56–83%, *p* < 0.001) regions than SPD children in the left foot during running, while the force in the Midfoot of SPD children is significantly larger than the healthy controls (during 29–57%, *p* < 0.001). As for the right foot, the healthy controls had larger forces in Toe 1 (during 8–26%, *p* < 0.001), Other Toes (during 19–22%, *p* = 0.045), M1 (during 23–26%, *p* < 0.01; and 63–72%, *p* = 0.001) and LatHeel (during 14–24%, *p* = 0.002), whilst the force in the Midfoot of SPD children is far greater than the healthy controls (during 6–100%, *p* < 0.001).

**Figure 11:**
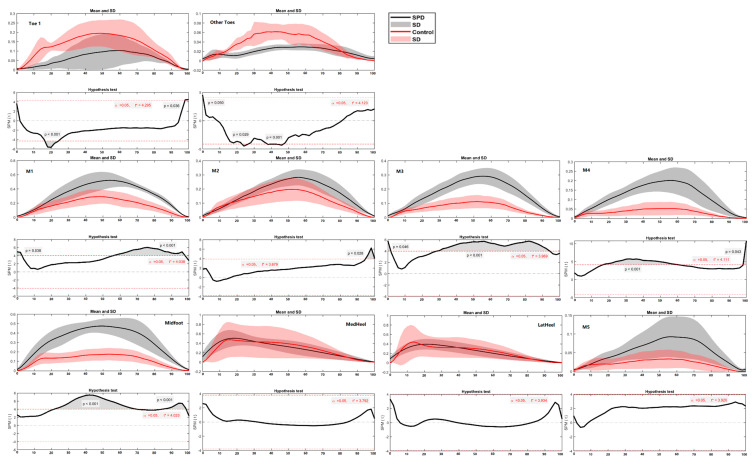
The regional plantar forces in the left foot during turning in the SPD children and healthy controls with highlighted statistics.

**Figure 12:**
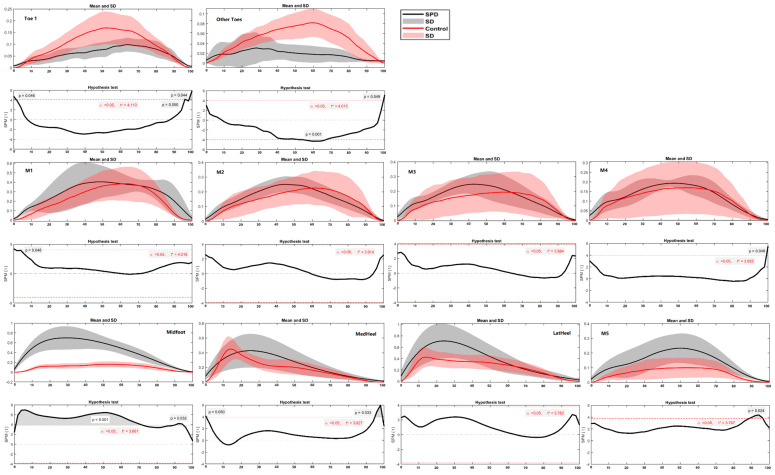
The regional plantar forces in the right foot during turning in the SPD children and healthy controls with highlighted statistics.

The main difference between SPD children and healthy controls was found in the toe, forefoot and midfoot regions (Figure 11 and Figure 12) during certain phases (in percentage) of the stance. Specifically, in the left foot while turning right, the difference was located at the starting and end phase of Toe 1. The healthy controls had larger force than the SPD children in Other Toes (during 56–66%, *p* = 0.001), while SPD children had greater force in Midfoot (during 1–70%, *p* < 0.001) than the healthy controls. As for the right foot while turning left, the healthy controls had larger forces in Toe 1 (during 16–24%, *p* < 0.001) and Other Toes (during 23–27%, *p* = 0.029; and 45–47%, *p* = 0.001), while SPD children had larger forces in M1 (during 57–97%, *p* < 0.001), M2 (during 94–100%, *p* = 0.028), M3 (during 1–3%, *p* = 0.046; and 30–94%, *p* < 0.001), M4 (during 17–56%, *p* < 0.001) and Midfoot (during 23–68%, *p* < 0.001; and 88–97%, *p* < 0.001).

## 4. Discussion

The purpose of this study was to compare differences in plantar loading and foot balance during the walking, running and turning activities in children with SPD via a comparative analysis with healthy controls in both left (non-dominant) and right (dominant) feet. The walking tasks had no significant difference between SPD children and healthy controls in both feet. During running, SPD children showed a supinated foot position during midstance with reduced plantar forces in the toes and metatarsals but focused forces in the midfoot of both feet. Similar loading patterns and foot COPs were found during turning where children with SPD had supinated foot posture during left turning and supination–pronation movement was restricted in both feet during turning. While compared to the healthy controls, the left foot had the most plantar force during right turning and the right foot had the most loading in the metatarsals and midfoot but less in toes during left turning among SPD children.

The current study found that the maximum force loading of the entire foot was around 1.5 BW during walking and 2–2.5 BW during running and turning activities, which were in accordance with the findings of previous studies [33,38,40]. A slightly higher sum force in the right limb might be due to limb dominance, as the non-dominant limb was responsible for support and stability, while the dominant limb played the role of propulsion [41]. The maximum force in the Midfoot region of SPD children in this study was around 0.7 BW, which was greater than the healthy controls in the range of previous studies [33,38]. This may be a key observation that should be noted, where children with SPD had reduced foot control during dynamic running and turning activities, while the healthy control group results were similar with the findings in the previous study, where age may affect the larger values of plantar force loadings [42]. Another information was that the children’s feet may be different to adult ones because of the plastic feature of the foot–ankle complex, especially in SPD children with the motor disorder of this study. The foot–ankle complex was often characterized by altered foot shape and function [43,44,45,46]. Because of the plasticity in this complex, the loading in the MF region increased greatly during running and turning, suggesting less stability or reduced control of the longitudinal arch [43] and reduced force in the distal segment of toes [47,48]. These could explain the larger MF (midfoot) loading but lesser toes loadings compared to the healthy controls in this study.

Assessments of the plantar loading and dynamic foot balance were employed to understand the foot structure and mechanics during dynamic conditions [49,50]. During walking, COP and FBI showed no significance between SPD children and healthy controls in both dominant and non-dominant feet. This may be because the SPD children of motor disorder cohort had stable behavioral automation [51] or less sensory hypersensitivity [6] in the neuromuscular system during preferred walking in a less dynamic condition.

However, the foot had restricted supination–pronation motions during running, showing a supinated COP trajectory and limited FBI range over the stance. This may be due to hypersensitivity from the neuroanatomical structure [6] in order to control motions of the foot–ankle complex with overprotection, thus restricting supination–pronation, which s reported as a key function for impact absorption [52]. This change was also linked with less plantar force in the medial and lateral heel regions of both feet, whilst the midfoot showed far greater force in both feet of SPD children compared to healthy controls. This difference may explain with the higher plasticity in the medial foot (arch region) during midstance [43]. Following the midstance was the push-off phase during running [43,44], which manifested with greater medial forefoot (M1, M2 and M3) force loadings in the healthy controls compared with the SPD children. The findings from this study may be useful, and it should be noted that SPD children had limited supination–pronation movement for shock attenuation in the foot complex and reduced ankle pronation to assist push-off and toe gripping movements.

During cutting, a more apparently limited motion in the foot–ankle complex was found, as shown in the COP trajectory and FBI of both feet compared with healthy controls. During the stance phase, most plantar loadings were exerted in the medial side of the stance foot with the foot in a pronated position among the healthy controls [53]. By nature, the COP trajectory transferred medially compared to the running in the healthy control, which also proved increasing foot pronation during the stance phase of turning. On the contrary, this transference was not observed in SPD children, and it still showed restricted supination–pronation motion. The finding suggested that sensory hypersensitivity may hinder the normal function in the foot–ankle complex, thus affecting balance and postural control during dynamic movement [6], such as the turning task of direction changes (or cutting maneuver). As documented in the literature, the youth population tended to employ ankle strategies to stay balance compared to the hip strategy of the aged population [21,54].

In this study, higher force loadings under M1 and M2 regions were found in both feet during turning, which agreed with a previous study [53]. The pressure load on the first metatarsal was positively associated with gait speed [55], suggesting that both SPD children and healthy controls had a similar range of run-turning gait speed. During turning activity (or known as cutting maneuvers), participants may decelerate in the original direction and redirect to the intended direction during turning, requiring acceleration [56]. Accordingly, the direction changing mechanism would account for the increased force loading under the M1 and M2 regions, and it is facilitated with active toe gripping motion in healthy controls (but not observed in SPD children) [46,48,57].

However, the significantly higher force profiles in the lateral forefoot of SPD children deserve special attention where active propulsive push-off occurred in these anatomical regions, which may result from reduced pronation motions. This alteration, thus, exposed SPD children into risks of falling and postural instability or ankle sprain during turning activities. In terms of force loadings in the midfoot regions, the reason was similar with running where there is high plasticity in the arch region. Turning is higher in terms of dynamics compared with running, which was also analyzed as a cutting maneuver in several specific sports such as soccer and basketball with recruited participants of college students or athletes. In this study, children with SPD were involved in order to be compared with healthy controls, which might be responsible for the different results. In this study, children with SPD more specifically had dyspraxia by conducting Sensory Integration and Praxis Tests [51]. The disorder in sensory processing might produce certain influences on motor behavior and disrupt the quality of movement [58].

Several limitations of current study should be considered. Firstly, this study only compared the difference in plantar loading, COP and FBI between SPD children and healthy control. The kinematic parameters and the muscle activities of the lower limb shall be analyzed to comprehensively investigate the movement characteristics of children with sensory processing disorder in future studies. Secondly, smaller numbers of participants were recruited in this study, which lacked generality to other age and gender cohorts, as this study was a pilot to reveal the movement patterns of this special SPD group. It was difficult to recruit more SPD children of different ages and genders, which shall be a primary aim in future study.

## 5. Conclusions

The current study aimed to compare plantar loading and foot balance during walking, running and turning activities in SPD children. No difference was found in foot balance and plantar loading during less dynamic walking, while limited supination–pronation motion was observed in SPD children during high dynamic running and turning. The plantar forces were mainly located in the midfoot region while less toes activity was found as well. It should be noted that SPD children had limited supination–pronation movement for shock attenuation in the foot complex and reduced ankle pronation to assist push-off and toe gripping movements. Understanding the behavior of plantar loading strategy and balance control during walking, running and turning activities may provide clinical implications for the rehabilitation and training of daily tasks.

## Data Availability

The data may be available upon reasonable request from the corresponding authors.

## Supplementary Materials

The following supporting information can be downloaded at the following website: https://www.mdpi.com/article/10.3390/children9030379/s1, Figure S1: The COP of trajectory in the left foot and right foot during walking with highlighted direction of pronation (Blue arrow) and supination (Red arrow); Figure S2: The foot balance index (FBI) in the left foot and right foot during walking with highlighted direction of pronation (blue arrow) and supination (red arrow); Figure S3: The vertical ground reaction force (GRF) in the left foot and right foot during walking in the SPD children and healthy controls; Figure S4: The regional plantar force in the left foot and right foot during walking in the SPD children and healthy controls.

## References

[1] Jussila K., Junttila M., Kielinen M., Ebeling H., Joskitt L., Moilanen I., Mattila M.L. (2020). Sensory Abnormality and Quantitative Autism Traits in Children with and without Autism Spectrum Disorder in an Epidemiological Population. J. Autism. Dev. Disord..

[2] Gentil-Gutiérrez A., Cuesta-Gómez J.L., Rodríguez-Fernández P., González-Bernal J.J. (2021). Implication of the sensory environment in children with autism spectrum disorder: Perspectives from school. Int. J. Environ. Res. Public Health.

[3] Molholm S., Murphy J.W., Bates J., Ridgway E.M., Foxe J.J. (2020). Multisensory Audiovisual Processing in Children With a Sensory Processing Disorder (I): Behavioral and Electrophysiological Indices Under Speeded Response Conditions. Front. Integr. Neurosci..

[4] Foxe J.J., Bene D., Ross V.A., Ridgway L.A., Francisco E.M., Molholm A.A. (2020). Multisensory Audiovisual Processing in Children With a Sensory Processing Disorder (II): Speech Integration Under Noisy Environmental Conditions. Front. Integr. Neurosci..

[5] Crasta J.E., Salzinger E., Lin M.H., Gavin W.J., Davies P.L. (2020). Sensory Processing and Attention Profiles Among Children With Sensory Processing Disorders and Autism Spectrum Disorders. Front. Integr. Neurosci..

[6] Ghanizadeh A. (2011). Sensory processing problems in children with ADHD, a systematic review. Psychiatry Investig..

[7] Ahn R.R., Miller L.J., Milberger S., McIntosh D.N. (2004). Prevalence of parents’ perceptions of sensory processing disorders among kindergarten children. Am. J. Occup. Ther..

[8] Ben-Sasson A., Carter A.S., Briggs-Gowan M.J. (2009). Sensory over-responsivity in elementary school: Prevalence and social-emotional correlates. J. Abnorm. Child. Psychol..

[9] Niutanen U., Harra T., Lano A., Metsäranta M. (2020). Systematic review of sensory processing in preterm children reveals abnormal sensory modulation, somatosensory processing and sensory-based motor processing. Acta Paediatr. Int. J. Paediatr..

[10] Miller L.J., Nielsen D.M., Schoen S.A., Brett-Green B.A. (2009). Perspectives on sensory processing disorder: A call for translational research. Front. Integr. Neurosci..

[11] Suarez H., Alonso R., Arocena S., Ferreira E., Roman C.S., Suarez A., Lapilover V. (2017). Sensorimotor interaction in deaf children. Relationship between gait performance and hearing input during childhood assessed in pre-lingual cochlear implant users. Acta Otolaryngol..

[12] Sheppard J., Young W. (2006). Agility literature review: Classifications, training and testing. J. Sports Sci..

[13] Dellal A., Keller D., Carling C., Chaouachi A., Wong D.P., Chamari K. (2010). Physiologic effects of directional changes in intermittent exercise in soccer players. J. Strength Cond. Res..

[14] Alexander R.M. (1991). Energy-saving mechanisms in walking and running. J. Exp. Biol..

[15] Cong Y., Lam W.K., Cheung J.T.M., Zhang M. (2014). In-shoe plantar tri-axial stress profiles during maximum-effort cutting maneuvers. J. Biomech..

[16] Glaister B.C., Orendurff M.S., Schoen J.A., Bernatz G.C., Klute G.K. (2008). Ground reaction forces and impulses during a transient turning maneuver. J. Biomech..

[17] Xu D., Carlton L.G., Rosengren K.S. (2004). Anticipatory postural adjustments for altering direction during walking. J. Mot. Behav..

[18] Xiang L., Mei Q., Fernandez J., Gu Y. (2020). A biomechanical assessment of the acute hallux abduction manipulation intervention. Gait Posture.

[19] Gao Z., Mei Q., Xiang L., Gu Y. (2020). Difference of walking plantar loadings in experienced and novice long-distance runners. Acta Bioeng. Biomech..

[20] Mei Q., Gu Y., Fernandez J. (2018). Alterations of Pregnant Gait during Pregnancy and Post-Partum. Sci. Rep..

[21] Mei Q., Feng N., Ren X.J.X., Lake M., Gu Y.D.Y.Y.D. (2015). Foot Loading Patterns With Different Unstable Soles Structure. J. Mech. Med. Biol..

[22] Hessert M.J., Vyas M., Leach J., Hu K., Lipsitz L.A., Novak V. (2005). Foot pressure distribution during walking in young and old adults. BMC Geriatr..

[23] Mei Q., Gu Y., Xiang L., Yu P., Gao Z., Shim V., Fernandez J. (2020). Foot shape and plantar pressure relationships in shod and barefoot populations. Biomech. Model. Mechanobiol..

[24] Lu Y.C., Mei Q.C., Gu Y.D. (2015). Plantar Loading Reflects Ulceration Risks of Diabetic Foot with Toe Deformation. Biomed. Res. Int..

[25] Hyun G.J., Jung T.W., Park J.H., Kang K.D., Kim S.M., Son Y.D., Cheong J.H., Kim B.N., Han D.H. (2016). Changes in Gait Balance and Brain Connectivity in Response to Equine-Assisted Activity and Training in Children with Attention Deficit Hyperactivity Disorder. J. Altern. Complement. Med..

[26] Lim B.O., O’Sull Ivan D., Choi B.G., Kim M.Y. (2016). Comparative gait analysis between children with autism and age-matched controls: Analysis with temporal-spatial and foot pressure variables. J. Phys. Ther. Sci..

[27] Nsenga Leunkeu A., Lelard T., Shephard R.J., Doutrellot P.L., Ahmaidi S. (2014). Gait cycle and plantar pressure distribution in children with cerebral palsy: Clinically useful outcome measures for a management and rehabilitation. NeuroRehabilitation.

[28] Chang W.D., Chang N.J., Lin H.Y., Lai P.T. (2015). Changes of plantar pressure and gait parameters in children with mild cerebral palsy who used a customized external strap orthosis: A crossover study. Biomed Res. Int..

[29] Provost B., Oetter P. (1994). The Sensory Rating Scale for Infants and Young Children: Development and Reliabity. Phys. Occup. Ther. Pediatr..

[30] Hilton C.L. (2011). Sensory Processing and Motor Issues in Autism Spectrum Disorders. International Handbook of Autism and Pervasive Developmental Disorders.

[31] Jorquera-Cabrera S., Romero-Ayuso D., Rodriguez-Gil G., Triviño-Juárez J.M. (2017). Assessment of sensory processing characteristics in children between 3 and 11 years old: A systematic review. Front. Pediatr..

[32] Chapman J.P., Chapman L.J., Allen J.J. (1987). The measurement of foot preference. Neuropsychologia.

[33] Gao Z., Mei Q., Xiang L., Baker J.S., Fernandez J., Gu Y. (2020). Effects of limb dominance on the symmetrical distribution of plantar loading during walking and running. Proc. Inst. Mech. Eng. Part P J. Sport. Eng. Technol..

[34] Mei Q., Xiang L., Li J., Fernandez J., Gu Y. (2021). Analysis of Running Ground Reaction Forces Using the One-Dimensional Statistical Parametric Mapping (SPM1d). J. Med. Biomech..

[35] Carpinella I., Crenna P., Calabrese E., Rabuffetti M., Mazzoleni P., Nemni R., Ferrarin M. (2007). Locomotor function in the early stage of Parkinson’s disease. IEEE Trans. Neural Syst. Rehabil. Eng..

[36] Willems T., Witvrouw E., Delbaere K., De Cock A., De Clercq D. (2005). Relationship between gait biomechanics and inversion sprains: A prospective study of risk factors. Gait Posture.

[37] Gao Z., Mei Q., Fekete G., Baker J.S., Gu Y. (2020). The Effect of Prolonged Running on the Symmetry of Biomechanical Variables of the Lower Limb Joints. Symmetry.

[38] Wen J., Ding Q., Yu Z., Sun W., Wang Q., Wei K. (2012). Adaptive changes of foot pressure in hallux valgus patients. Gait Posture.

[39] Pataky T.C. (2012). One-dimensional statistical parametric mapping in Python. Comput. Methods Biomech. Biomed. Eng..

[40] Saxby D.J., Modenese L., Bryant A.L., Gerus P., Killen B., Fortin K., Wrigley T.V., Bennell K.L., Cicuttini F.M., Lloyd D.G. (2016). Tibiofemoral contact forces during walking, running and sidestepping. Gait Posture.

[41] Sadeghi H., Allard P., Prince F., Labelle H. (2000). Symmetry and limb dominance in able-bodied gait: A review. Gait Posture.

[42] Mesquita P.R., Neri S.G.R., Lima R.M., Manfio E.F., De David A.C. (2019). Running and walking foot loading in children aged 4–10 years. J. Appl. Biomech..

[43] Bosch K., Gerß J., Rosenbaum D. (2010). Development of healthy children’s feet-Nine-year results of a longitudinal investigation of plantar loading patterns. Gait Posture.

[44] Alvarez C., De Vera M., Chhina H., Black A. (2008). Normative data for the dynamic pedobarographic profiles of children. Gait Posture.

[45] Shu Y., Mei Q., Fernandez J., Li Z., Feng N., Gu Y. (2015). Foot Morphological Difference between Habitually Shod and Unshod Runners. PLoS ONE.

[46] Mei Q., Fernandez J., Fu W., Feng N., Gu Y. (2015). A comparative biomechanical analysis of habitually unshod and shod runners based on a foot morphological difference. Hum. Mov. Sci..

[47] Mei Q., Gu Y., Fernandez J. (2019). A biomechanical assessment of running with hallux unstable shoes of different material stiffness. Acta Bioeng. Biomech..

[48] Lambrinudi C. (1932). Use and Abuse of Toes. Postgrad. Med. J..

[49] Nawata K., Nishihara S., Hayashi I., Teshima R. (2005). Plantar pressure distribution during gait in athletes with functional instability of the ankle joint: Preliminary report. J. Orthop. Sci..

[50] Chuckpaiwong B., Nunley J.A., Mall N.A., Queen R.M. (2008). The effect of foot type on in-shoe plantar pressure during walking and running. Gait Posture.

[51] Koziol L.F., Budding D.E., Chidekel D. (2011). Sensory integration, sensory processing, and sensory modulation disorders: Putative functional neuroanatomic underpinnings. Cerebellum.

[52] Nigg B., Behling A.V., Hamill J. (2019). Foot pronation. Footwear Sci..

[53] Orendurff M.S., Rohr E.S., Segal A.D., Medley J.W., Green J.R., Kadel N.J. (2008). Regional foot pressure during running, cutting, jumping, and landing. Am. J. Sports Med..

[54] Best A.N., Wu A.R. (2020). Upper body and ankle strategies compensate for reduced lateral stability at very slow walking speeds. Proc. R. Soc. B Biol. Sci..

[55] Segal A., Rohr E., Orendurff M., Shofer J., O’Brien M., Sangeorzan B. (2004). The effect of walking speed on peak plantar pressure. Foot Ankle Int..

[56] Havens K.L., Sigward S.M. (2015). Cutting mechanics: Relation to performance and anterior cruciate ligament injury risk. Med. Sci. Sports Exerc..

[57] Mei Q., Fernandez J., Hume P., Gu Y. (2016). Investigating biomechanical function of toes through external manipulation integrating analysis. Acta Bioeng. Biomech..

[58] Lane S.J., Mailloux Z., Schoen S., Bundy A., May-Benson T.A., Parham L.D., Roley S.S., Schaaf R.C. (2019). Neural foundations of ayres sensory integration. Brain Sci..

