# Supplementary material for: Understanding Foot Loading and Balance Behavior of Children with Motor Sensory Processing Disorder
Source: Children (Basel). 2022 Mar 9;9(3):379. doi: 10.3390/children9030379 (PMC8947083; doi:10.3390/children9030379)
Supplement: Supplementary file 1 [file children-09-00379-s001.zip › children-1598332-supplementary.pdf]

Analysis of Task-based Plantar Loadings and Foot Balance in Children with Sensory Processing Disorders

Lin Yu [1,2], Peimin Yu [1,3,4], Wei Liu [1,5], Zixiang Gao [1,5], Dong Sun [6,*], Qichang Mei [1,3,4,*], Justin Fernandez [1,3,4] and Yaodong Gu [1,3,4,*]

Supplementary materials

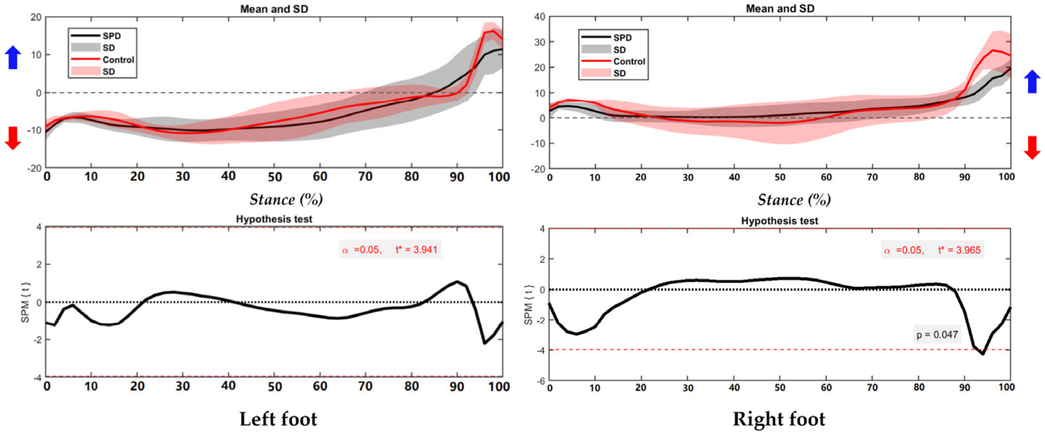

Figure S1. The COP of trajectory in the left foot and right foot during walking with highlighted direction of pronation (Blue arrow) and supination (Red arrow)

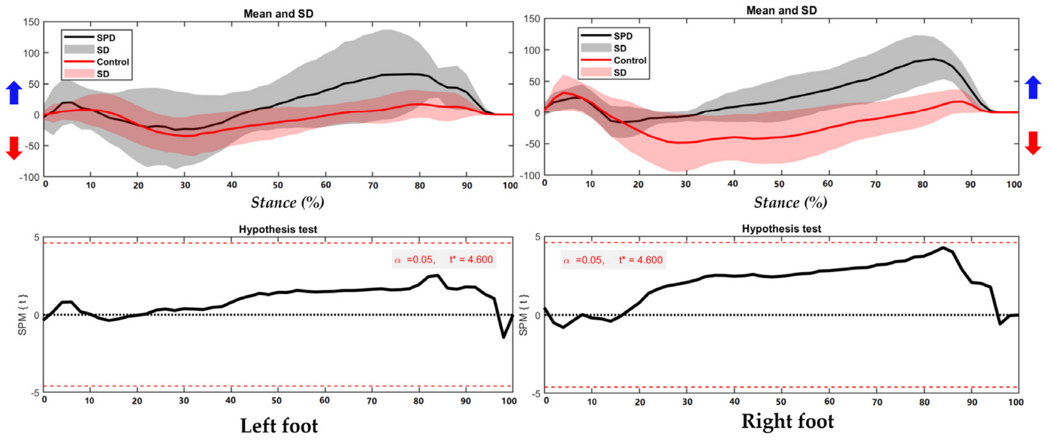

Figure S2. The foot balance index (FBI) in the left foot and right foot during walking with highlighted direction of pronation (Blue arrow) and supination (Red arrow)

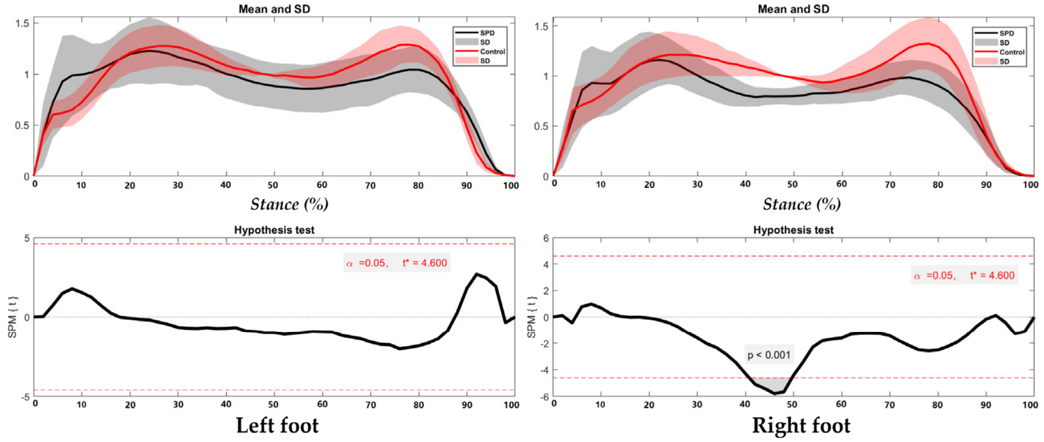

Figure S3. The vertical ground reaction force (GRF) in the left foot and right foot during walking in the SPD children and healthy controls

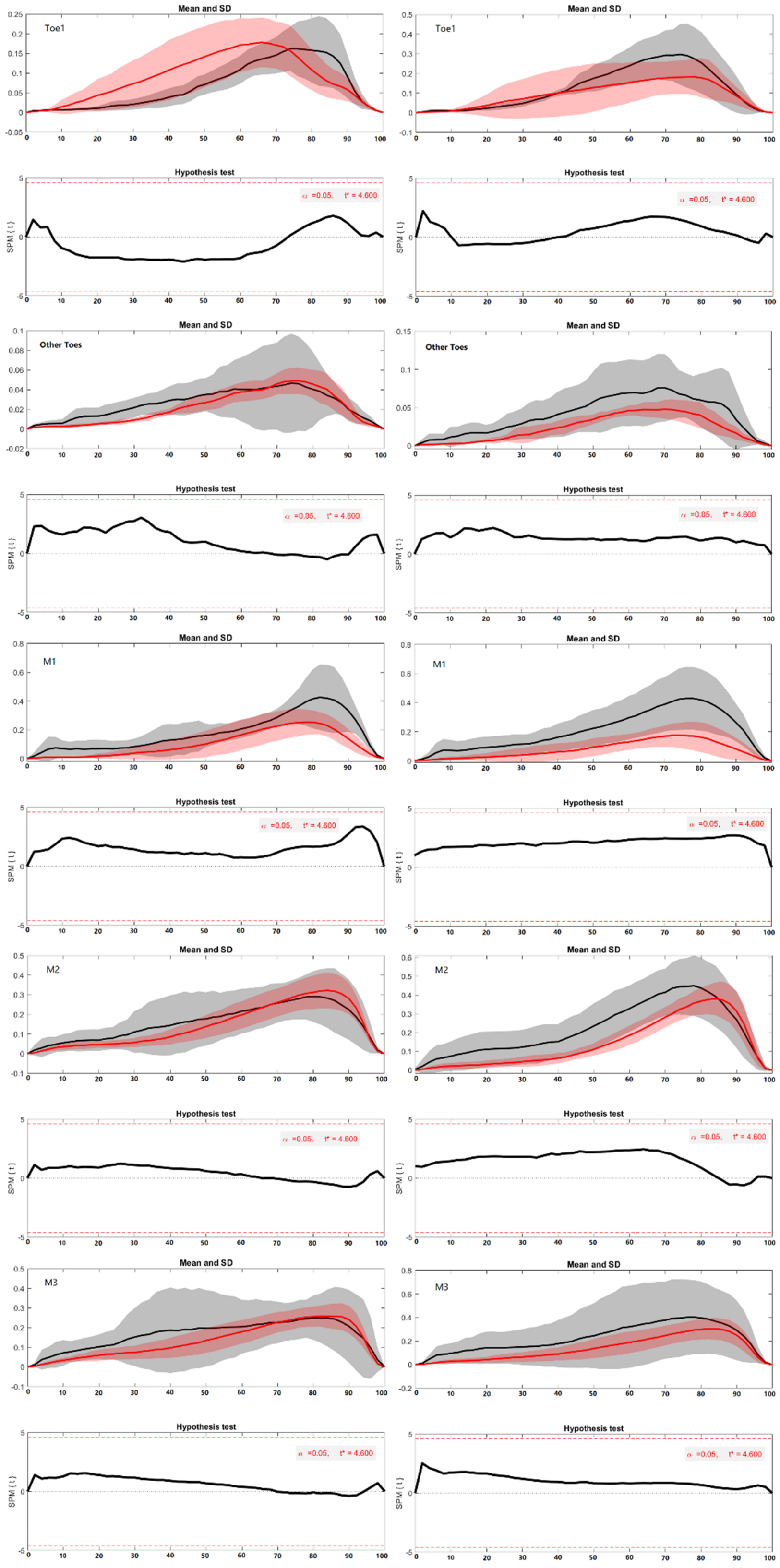

Figure S4-1. The regional plantar force in the left foot and right foot during walking in the SPD children and healthy controls

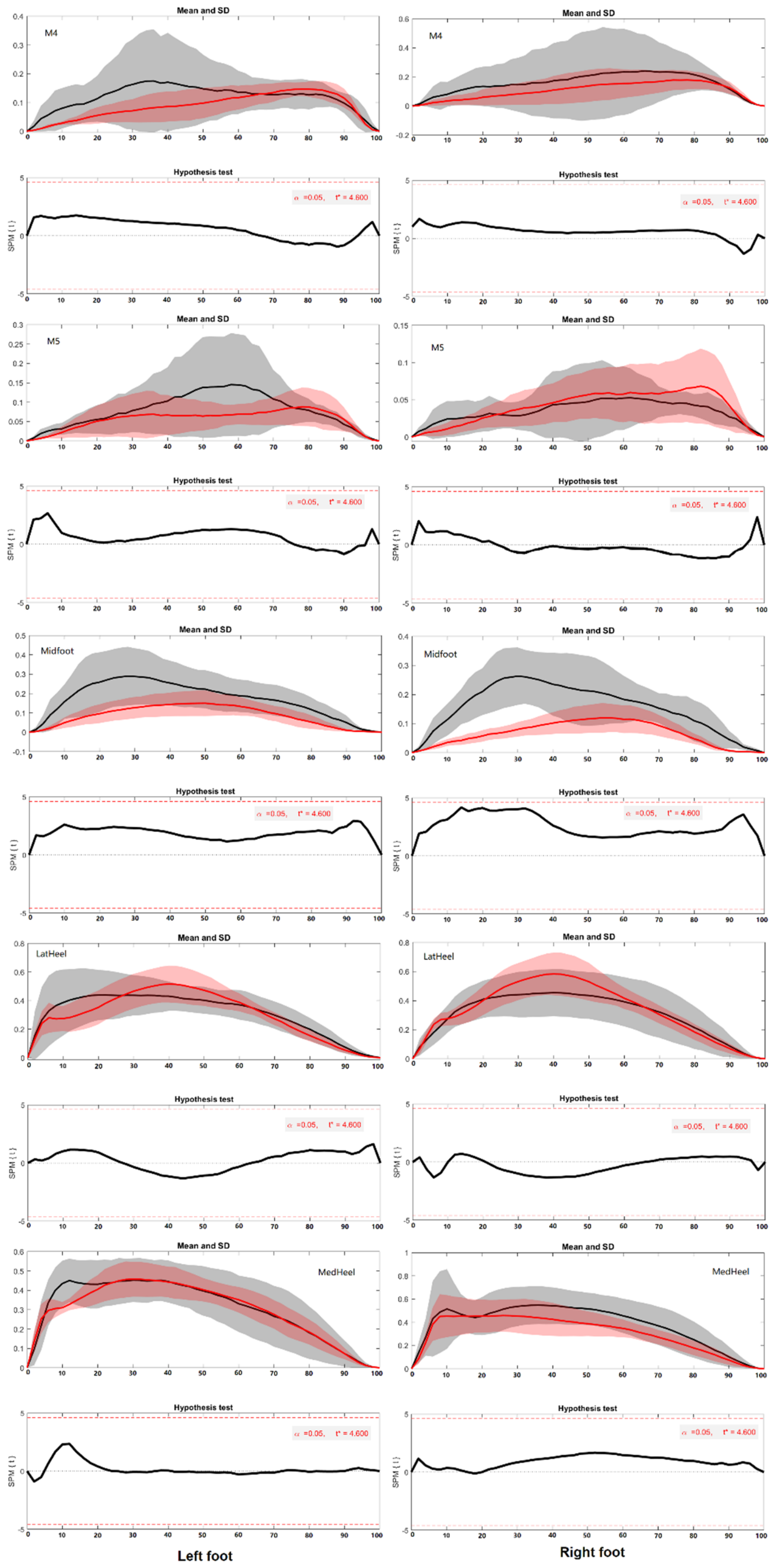

Figure S4-2. The regional plantar force in the left foot and right foot during walking in the SPD children and healthy controls
